# Health Information and Misinformation: A Framework to Guide Research and Practice

**DOI:** 10.2196/38687

**Published:** 2023-06-07

**Authors:** Ilona Fridman, Skyler Johnson, Jennifer Elston Lafata

**Affiliations:** 1 Lineberger Comprehensive Cancer Center University of North Carolina Chapel Hill, NC United States; 2 Radiation Oncology Deparment Huntsman Cancer Hospital University of Utah Utah, UT United States; 3 Eshelman School of Pharmacy University of North Carolina Chapel Hill, NC United States

**Keywords:** misinformation, social networks, decision-making, information validation, policy, health information, web-based

## Abstract

When facing a health decision, people tend to seek and access web-based information and other resources. Unfortunately, this exposes them to a substantial volume of misinformation. Misinformation, when combined with growing public distrust of science and trust in alternative medicine, may motivate people to make suboptimal choices that lead to harmful health outcomes and threaten public safety. Identifying harmful misinformation is complicated. Current definitions of misinformation either have limited capacity to define harmful health misinformation inclusively or present a complex framework with information characteristics that users cannot easily evaluate. Building on previous taxonomies and definitions, we propose an information evaluation framework that focuses on defining different shapes and forms of harmful health misinformation. The framework aims to help health information users, including researchers, clinicians, policy makers, and lay individuals, to detect misinformation that threatens truly informed health decisions.

## Introduction

Almost 3 quarters of people (72%) use the internet first when they need health-related information [1]. Web-based information helps people to prepare for conversations with clinicians, facilitates self-care, and improves adherence to physicians’ advice and recommended medication use [2]. However, the benefits of web-based information come with challenges. To find credible information, individuals often need to sort through misinformation, which may include posts about potentially harmful practices, unproven alternative therapies, pseudoscientific explanations, rumors, and misappropriations [3,4]. Misinformation, in fact, has an overwhelmingly high prevalence—up to 40% of posts on social media contain health misinformation related to vaccinations; eating disorders; treatments; and chronic diseases, including cancer [5].

Health misinformation could mislead health-related decisions and result in harmful outcomes. A recent physician evaluation of popular social media posts found frequent health misinformation and identified that almost a third (31%) of such posts could lead to individuals delaying standard treatment or engaging in potentially toxic, expensive, and futile therapies [6]. Decisions driven by misinformation can lead to emotional damage, false hopes, financial loss, and more importantly, physical damage that hastens death [7-9]. Although a comprehensive evaluation of the negative effect of misinformation on patient outcomes has not been completed, multiple case reports describe individuals who have suffered negative consequences after they followed web-based misinformation [10], including prominent cases with public figures, such as Steve Jobs [11] and William Hurt [12]. Perhaps the most devastating effect of misinformation is that it sows doubt in medical science. In extreme cases, such doubts can lead to social movements advocating decisions that threaten public safety. For instance, motivated by misinformation that was spread by antivaccine supporters, a substantial proportion of people in the United States chose not to receive vaccines against the COVID-19 virus despite their proven safety and effectiveness [13,14].

To date, no comprehensive system can reliably detect and neutralize harmful health misinformation, partially because harmful misinformation takes multiple shapes and forms. More than 50 distinct types of misinformation are described in the literature, such as fake news, manipulation, rumors, fabrication, and click bites [15-17]. The most common definitions of misinformation are developed based on a single information characteristic, such as truthfulness or author motivation (disinformation) [18,19], including two definitions specifically related to health misinformation [20,21]. As a result, certain types of harmful health misinformation are not covered by these definitions. For instance, one of the most common definitions suggests that misinformation is information that contradicts truthful facts, where truth is defined as a fact or opinion that is aligned with the expert consensus or the best scientific evidence available at that time [18]. This definition does not cover cases in which truthful facts are exaggerated, misinterpreted, or used in the wrong context. For instance, SanSentinel [22] distributed a story about a physician dying after receiving a COVID-19 vaccination. The chronology of the events was truthfully described in the article. However, the connection between the physician's vaccination and death was never established. Despite the cause of death not being verified, the news ignited a misinformed public discussion about the dangers of vaccination. The story reached almost 50 million views on Facebook [23]. Some proportion of those individuals who viewed the Facebook message were likely motivated to reject or delay vaccination, which, in turn, prolonged the damage of COVID-19 to public health.

More inclusive definitions usually consist not of one but a composite of information characteristics. However, frequently, these characteristics are not considered from a user point of view and may be challenging to evaluate. For instance, author motivation is a common characteristic that is used in misinformation definitions. The core issue is that authors could be motivated by a mixture of positive, negative, and selfish interests. For example, an author could have financial interests in posting an advertisement for medication with unknown outcomes but also may genuinely intend to help treat a condition. In this and other similar situations, author motivation is difficult to discern, even for experts in the field.

The overarching purpose of this viewpoint is to propose a composite framework that covers the substantial proportion of harmful health misinformation but is simple enough to be applied by health information users, including researchers, clinicians, policy makers, and lay individuals. The development of the framework is guided by the practical goal of helping users identify and prevent the negative impact of misinformation on decisions related to various aspects of health, including preventive medicine, therapeutic care, and lifestyle behaviors. Therefore, we focused the framework on misinformation that has the potential to cause harm to health-related decisions, inclusive of physical, emotional, social, and financial harm.

## Misinformation Characteristics

The characteristics of misinformation are defined in this framework as abstract rules that can be used to judge the quality of information [24]. We used 3 criteria to suggest the characteristics of misinformation that could be helpful in detecting harmful health misinformation. First, characteristics should be observable. In other words, a user should be able to evaluate a characteristic on their own or in consultation with an expert (clinician). As alluded to above, motivation tends to be an unobservable characteristic. Second, information characteristics should be generalizable across multiple contexts and media. Taxonomies and examples specific to media (eg, click bites) were not included. Third, characteristics of information should be simple. Thus, characteristics that contained branching logic and subcategories were excluded. According to these criteria, we chose the following key characteristics of misinformation for the framework: actionability, verifiability, and facticity. The examples of misinformation taxonomies that we used to choose misinformation characteristics are provided in Table 1 [15-17,25-34].

**Table 1:** Summary of the characteristics of misinformation.

Articles	Characteristics of misinformation as identified by the authors	Reasons for not including some characteristics
Kapantai et al [15], 2020	Motivation, verifiability, and facticity	Observability: motivation or intention
Southwell et al [25], 2019	Actionability and audience exposure	Observability: audience exposure
Tandoc et al [26], 2018	Level of facticity and authors’ intention to deceive	Observability: motivation or intention
Zannottou et al [17], 2019	Types of misinformation (eg, fabrication and propaganda) and motivation	Generalizability: types of misinformation; Observability: motivation or intention
Kumar et al [27], 2018	Opinion based (eg, fake reviews), fact based, and with intention to deceive	Observability: opinion-based information (fake reviews) as well as motivation or intention
Gabarron et al [28], 2021	Myths, sarcasm, and humor	Generalizability: types of misinformation
Jamison et al [29], 2020	Antivaccine conspiracies and provaccine promotions	Generalizability: specific context
Paquin et al [30]^a^, 2022	True claim, misleading claim (ie, implicit misinformation), and false claim (ie, explicit misinformation)	Observability: implicit misinformation
Wardle et al [31], 2017	Disinformation (false information to harm), misinformation (false information), and malinformation (true information that is used to harm)	Observability: disinformation and malinformation
Lemieux et al [32], 2018	Inaccuracy, unreliability, and inauthenticity	Simplicity: unreliability and inauthenticity
Dhoju et al [33], 2019	Reliable media and unreliable media	Generalizability: type of media
Molina et al [16], 2021	Real news, fake news, commentary (opinion), misreporting (accidentally not true), polarized and sensationalist content, citizen journalism, satire, and persuasive information	Generalizability: type of article
Wang et al [34], 2022	Intentions, perception of the information or relevance^a^, benchmarks of facticity, and scope	Observability: motivation or intention as well as scope

^a^Perception of information is defined as the perceived usefulness of information in a problem-solving information search. We interpret this concept as whether users perceive information as worth acting upon; in other words, whether they evaluate information as actionable.

### Actionability

Actionability of information is defined by whether the information can lead a person to change their attitude or action (doing or not doing something), which they would not have done without learning the information. One could evaluate actionability by considering to what extent the information is useful for solving a specific health problem [35]. Not all information is actionable [25,35]. In some cases, the actionability of information is defined by users’ perspectives. Information might motivate behavior change among some populations but not others. For instance, messages related to screening for sex-related cancers, such as breast or prostate cancer, may not be relevant for health information users of the opposite sex. Similarly, misinformation about medication related to heart diseases [36] might be judged as actionable by older populations more than younger populations.

In other cases, actionability of information is defined by the nature of information. Certain types of information might be irrelevant for health-related problems. An example of such information might be a hoax disclosing a cancer diagnosis by a celebrity [37]. Without a further discussion of the celebrity’s previous lifestyle or medical choices, this information is nonactionable. Other examples could be honest errors in attributing information to a wrong source [25] or some forms of click bites, which are attractive titles that are not supported by information in the text. The misleading titles could be debunked when one engages in reading the article [19].

Actionable information may contain a direct call for action, including recommendations to buy medication; engage in therapy; change diets and lifestyle behaviors; or repost the information itself. Actionable information could hide in opinions and personal stories. A notable example is the story of Belle Gibson. In her web-based blog, she disclosed her experience of treating brain cancer with ayurvedic medicine, oxygen therapy, as well as a gluten and sugar-free diet [38]. She claimed to reach a complete cure via these actions. Before it became known that she had faked her diagnosis, she built a profitable business selling futile dieting as a cancer cure to her followers [39]. Not only personal stories but also simple opinion statements may have a dramatic effect on public health. For instance, at the beginning of the COVID-19 pandemic, President Donald Trump stated that people have a choice whether to wear masks for protection; he also claimed that he personally decided not to wear a mask. According to the epidemiological model proposed by researchers from Emory University, if the President’s statement reduced mask use by 25%, it caused 4244 deaths in the United States alone [40].

As such, we propose that health information users sort information based on whether the information prompts them to change attitudes or take a particular action with regard to solving a health-related problem. Evaluation of actionability could reduce the cognitive load of information evaluation, allowing users to ignore nonactionable information while beware of the influence hidden in personal stories and opinions. If information users detect that the information is likely to result in behavior or attitude change, the information needs to be flagged for further assessment of facticity.

### Facticity

Facticity is formally defined by whether the information is consistent with the evidence or consensus of the scientific community at the time of evaluation [18]. Factual information usually originates from data, scientific reports, rigorous clinical trials, observational studies, or documented agreements of field experts. Facticity is a key component that underlines identifying harmful information. Decisions that are based on nonfactual information have unknown, and at times, harmful outcomes. For individuals with medical conditions and those who receive standard medical therapies, this path is especially precarious. Some complementary supplements, diets, and alternative therapies may not be harmful when used independently but may become toxic in combination with standard therapies [41].

Multiple recommendations have been developed to guide health information users in their evaluation of information facticity [42-46]. Although recommendations vary in complexity, the majority of them ask users to do the following:

- Identify authors and their credentials
- Understand authors’ conflicts of interest
- Learn about funding sources
- Identify and evaluate original sources of information
- Compare information among different sources
- Determine the date of posting

The evaluation of facticity is an arduous task. First, many health information users might not be equipped to implement some of the recommended steps. For instance, the recommendation “evaluation of original sources” may require users to have some scientific knowledge in interpreting data and expertise in determining the quality of scientific reports. The second challenge is that information frequently presents a mixture of true and false statements that occur due to honest errors, misunderstanding, and sometimes because of authors’ motivated intentions. For instance, a recent news report stated that “a vaccine wiped out cancer from a patient” [47]. The report described a clinical trial that enrolled patients with breast cancer and a patient who stated that her cancer was gone. The report delivered partially truthful information. A clinical trial for vaccination against breast cancer is ongoing, but the conclusion about the effectiveness of the vaccine was premature and false. In fact, several years of surveillance are required before the effectiveness of this vaccine can be reported [48]. Such partially factual reports may motivate patients’ decisions, which will likely result in financial loss, false hopes, and disappointment. The third challenge is that facticity might change over time if new scientific evidence becomes available and alters the balance of benefits and harms [18]. For instance, a medication for hypertension, Mibefradil (Posicor), was approved as effective and safe. Later, it was discovered that in combination with other medications, it increased the risk of death. According to some sources, Mibefradil caused more than 100 deaths before it was recalled [49].

Although complex, establishing facticity is an important task for health information users, which needs to be conducted continuously due to the possibility of changes in scientific evidence. If the evidence is established or consensus among experts is reached, facticity could be determined [18]. However, if evidence and experts’ opinions remain emergent or are controversial, it is difficult to establish facticity. In this case, we suggest that the information should be flagged as unverifiable.

### Verifiability

Verifiability is a characteristic of information that is defined by the availability of evidence or scientific agreement that could support a piece of information. Whether information is verifiable could be established during facticity evaluation, although some types of information may be judged as unverifiable preemptively. Such types of information range from personal stories to articles describing newly discovered “breakthrough” medicine, for which rigorous scientific studies have not been conducted.

Personal stories on social media and patient testimonies are common examples of unverifiable health information. Health information users might find personal stories helpful because stories allow them to learn medical terminology, visualize different processes of treatment, and understand how side effects feel [50]. However, personal stories could not be reliably verified, as the author might fake the diagnosis or describe a unique rare case that falls outside the scientific evidence, and therefore, will not be relevant to other patients’ experiences.

Flagging information as unverifiable could help health information users to assign a lesser weight to such information when a decision needs to be made, remain doubtful and open-minded about the subject, and adjust their decisions if an expert’s opinion or new evidence becomes available. If unverifiable information needs to be used to inform health-related decisions, health information users need to treat it as nonfactual and take necessary precautionary steps, such as careful estimation of potential harms and benefits as well as thorough consultation with clinical experts.

## Framework for Defining Harmful Health Misinformation

The challenge of misinformation is a daunting one, and unfortunately, it is a problem that is here to stay. With the advent of social media and the ease of sharing web-based information, false and misleading health information spreads rapidly and has significant consequences for public health. Despite the ongoing efforts of researchers, public health officials, and technology companies, misinformation continues to persist and is becoming increasingly difficult to combat. This complex issue requires a multifaceted approach involving education, technology, and policy interventions. To create effective strategies and mitigate the negative impacts of misinformation, we must prioritize interventions that are both evidence-based and realistically implementable. This requires a systematic approach that includes classifying different types of misinformation. Gaining a comprehensive understanding of the various manifestations of misinformation enables us to develop targeted interventions that systematically address persistent issues and effectively curtail the dissemination of false or harmful information.

**Figure 1:**
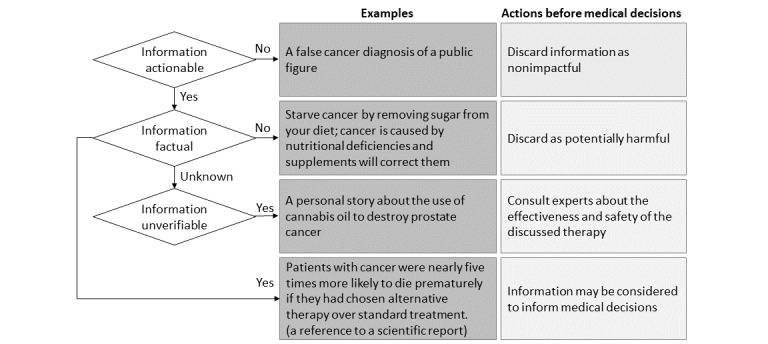
Health information classification.

The framework presented in Figure 1 is designed to assist health information users in classifying information and guide them on how to approach verifying health information that could mislead their decisions. The framework focuses on 3 characteristics of information: actionability, facticity, and verifiability. If something is not actionable, it may be considered unimportant and can be discarded. Facticity is an essence that information users aim to achieve. However, identifying facticity can be challenging, and in some cases, it may be impossible due to the lack of available evidence or knowledge. Therefore, the third component—unverifiability—is included in the framework. To address unverifiable information effectively, it is recommended to seek expert opinions on the potential risks associated with the information. In contrast to other frameworks, our approach is founded on the principle of observability and strikes a balance between comprehensiveness and simplicity.

Thus, this framework is user-friendly and could be applied by various stakeholders to combat health misinformation. For instance, individual users can learn from the framework that if they are unsure about the accuracy of information, they should label it as unverifiable and seek expert opinion instead of continuing to search for more information, which may lead only to confusion or false confidence. Researchers developing algorithmic detection of misinformation can flag both nonfactual and unverifiable information to safeguard health information users from futile verification attempts. Clinicians can use the framework during patient encounters to initiate conversations on how to approach information evaluation and identify harmful misinformation. They can encourage patients to consider not only facticity but also information’s actionability and verifiability to help patients prioritize the strategies of information vetting. Further, they could emphasize the uncertainty of outcomes behind unverifiable information to ensure that patients make truly informed decisions. With this framework, policy makers are better equipped to introduce the concept of uncertainty behind scientific evidence that informs public health policies. Specifically, policy makers can provide clarifications on which aspects of information should be deemed actionable and which aspects are currently unverifiable. The approach will enable the public to remain receptive and amend their decisions in response to new evidence. Overall, the framework aims to unite health information users, researchers, clinicians, and policy makers in their effort to develop a comprehensive system that helps detect and combat health-related misinformation. This systematic approach enables us to create a more informed and empowered society, one that is better equipped to identify and combat the negative effects of health misinformation.

## References

[1] Health Information National Trends Survey (HINTS).

[2] Thapa DK, Visentin DC, Kornhaber R, West S, Cleary M (2021). The influence of online health information on health decisions: a systematic review. Patient Educ Couns.

[3] Wang Y, McKee M, Torbica A, Stuckler D (2019). Systematic Literature Review on the Spread of Health-related Misinformation on Social Media. Soc Sci Med.

[4] Afful-Dadzie E, Afful-Dadzie A, Egala S (2023). Social media in health communication: a literature review of information quality. Health Inf Manag.

[5] Suarez-Lledo V, Alvarez-Galvez J (2021). Prevalence of health misinformation on social media: systematic review. J Med Internet Res.

[6] Johnson SB, Parsons M, Dorff T, Moran MS, Ward JH, Cohen SA, Akerley W, Bauman J, Hubbard J, Spratt DE, Bylund CL, Swire-Thompson B, Onega T, Scherer LD, Tward J, Fagerlin A (2021). Cancer misinformation and harmful information on Facebook and other social media: a brief report. J Natl Cancer Inst.

[7] Johnson S, Park H, Gross C, Yu J (2018). Use of alternative medicine for cancer and its impact on survival. J Natl Cancer Inst.

[8] Lau AY, Gabarron E, Fernandez-Luque L, Armayones M (2012). Social media in health--what are the safety concerns for health consumers?. Health Inf Manag.

[9] Johnson SB, Park HS, Gross CP, Yu JB (2018). Complementary medicine, refusal of conventional cancer therapy, and survival among patients with curable cancers. JAMA Oncol.

[10] Crocco AG, Villasis-Keever M, Jadad AR (2002). Analysis of cases of harm associated with use of health information on the internet. JAMA.

[11] Walton A (2011). Steve Jobs's Cancer Treatment Regrets. Forbes.

[12] (2018). Actor William Hurt vouches for side effect-free cancer therapy at unveiling. CBC News.

[13] Monte LM (2021). Household pulse survey shows many don't trust COVID vaccine, worry about side effects. US Census Bureau.

[14] CDC COVID-19 Response Team (2021). SARS-CoV-2 B.1.1.529 (Omicron) variant - United States, December 1-8, 2021. MMWR Morb Mortal Wkly Rep.

[15] Kapantai E, Christopoulou A, Berberidis C, Peristeras V (2020). A systematic literature review on disinformation: toward a unified taxonomical framework. New Media & Society.

[16] Molina MD, Sundar SS, Le T, Lee D (2019). “Fake news” is not simply false information: a concept explication and taxonomy of online content. ABS.

[17] Zannettou S, Sirivianos M, Blackburn J, Kourtellis N (2019). The web of false information. J Data and Information Quality.

[18] Vraga EK, Bode L (2020). Defining misinformation and understanding its bounded nature: using expertise and evidence for describing misinformation. Political Communication.

[19] (2018). A multi-dimensional approach to disinformation : report of the independent high level group on fake news and online disinformation. European Commission.

[20] Lazer David, Swire-Thompson (2020). Public health and online misinformation: challenges and recommendations. Annu Rev Public Health.

[21] Chou WS, Oh A, Klein WMP (2018). Addressing health-related misinformation on social media. JAMA.

[22] Boryga A (2021). A ‘healthy’ doctor died two weeks after getting a COVID-19 vaccine; CDC is investigating why. SunSentiel.

[23] Alba D, Mac R (2021). Facebook, fearing public outcry, shelved earlier report on popular posts. The New York Times.

[24] Zhang Y, Sun Y, Xie B (2015). Quality of health information for consumers on the web: a systematic review of indicators, criteria, tools, and evaluation results. J Assn Inf Sci Tec.

[25] Southwell BG, Niederdeppe J, Cappella JN, Gaysynsky A, Kelley DE, Oh A, Peterson EB, Chou WS (2019). Misinformation as a misunderstood challenge to public health. Am J Prev Med.

[26] Tandoc EJ, Lim Z, Ling R (2018). Fake news. Digit Journal.

[27] Kumar S, Shah N False information on web and social media: a survey. arXiv.

[28] Gabarron E, Oyeyemi SO, Wynn R (2021). COVID-19-related misinformation on social media: a systematic review. Bull World Health Organ.

[29] Jamison A, Broniatowski DA, Smith MC, Parikh KS, Malik A, Dredze M, Quinn SC (2020). Adapting and extending a typology to identify vaccine misinformation on Twitter. Am J Public Health.

[30] Paquin R, Boudewyns V, Betts K, Johnson M, O?Donoghue A, Southwell B (2022). An empirical procedure to evaluate misinformation rejection and deception in mediated communication contexts. Commun Theory.

[31] Wardle C, Derakhshan H Information disorder: toward an interdisciplinary framework for research and policymaking. Council of Europe.

[32] Lemieux V, Smith T (2018). Leveraging archival theory to develop a taxonomy of online disinformation.

[33] Dhoju S, Rony M, Kabir M, Hassan N (2019). A large-scale analysis of health journalism by reliable and unreliable media. Stud Health Technol Inform.

[34] Wang Y, Thier K, Nan X, Keselman A, Smith CA, Wilson A (2022). Defining health misinformation. Combating Online Health Misinformation: A Professionals Guide to Helping the Public.

[35] Krishna A, Thompson T (2019). Misinformation about health: a review of health communication and misinformation scholarship. American Behavioral Scientist.

[36] Martin SS 3 myths about cholesterol-lowering statin drugs. John Hopkins Medicine.

[37] Rumer A (2018). Kim Zolciak Biermann Denies Lying About Cancer Diagnosis. Popculture.

[38] (2021). Belle Gibson: the influencer who lied about having cancer. BBC.

[39] (2014). Melbourne mum Belle Gibson on taking the world by storm with her app The Whole Pantry, while fighting terminal brain cancer. The Globe and Mail.

[40] Hahn RA (2021). Estimating the COVID-related deaths attributable to President Trump's early pronouncements about masks. Int J Health Serv.

[41] (2022). Complementary and alternative medicine. National Cancer Institute.

[42] (2015). How to find cancer resources you can trust. National Cancer Institute.

[43] Health misinformation. US Department of Health and Human Services.

[44] (2021). What to know when searching for cancer information online: an expert perspective. Cancer.Net.

[45] Finding cancer information on the internet. American Cancer Society.

[46] Health on the Net (HON).

[47] Orlando F3 (2019). Trial vaccine wipes out breast cancer in Florida patient. FOX 10 Phoenix.

[48] Pallerla S, Abdul ARM, Comeau J, Jois S (2021). Cancer vaccines, treatment of the future: with emphasis on HER2-positive breast cancer. Int J Mol Sci.

[49] Stolberg S (1998). Heart drug withdrawn as evidence shows it could be lethal. The New York Times.

[50] Harkin LJ, Beaver K, Dey P, Choong K (2017). Navigating cancer using online communities: a grounded theory of survivor and family experiences. J Cancer Surviv.

